# Assessing Wear Characteristics of Sprayable, Diacetylene-Containing Sensor Formulations

**DOI:** 10.3390/s24216925

**Published:** 2024-10-29

**Authors:** Priyanka Shiveshwarkar, Anthony David Nelson, My Thi Nguyen, Justyn Jaworski

**Affiliations:** Department of Bioengineering, The University of Texas at Arlington, 500 UTA Blvd., Arlington, TX 76010, USA; priyanka.shiveshwarkar@mavs.uta.edu (P.S.); anthony.nelson@uta.edu (A.D.N.); mtn0381@mavs.uta.edu (M.T.N.)

**Keywords:** sprayable sensors, diacetylene, wear resistance, environmental monitoring

## Abstract

This work extends recent developments in diacetylene-based, sprayable sensors by identification and assessment of formulations which facilitate their use for wearable sensing. Diacetylene-based spray-on sensors have the potential to be a widely deployed sensing technology, as they require no power and can be applied as thin coatings onto surfaces to provide a colorimetric response to target exposure. In responding to radiation, liquid-phase targets, or gas-phase targets specifically determined by the formulation of the sprayable sensor used, this technology is amenable to wearable sensors for measuring exposure to different environmental risks. Here, we provide the means to improve wear resistance, reduce false-positive signals due to wetting, and enhance color fastness for coatings of sprayable, diacetylene-based sensor formulations on cotton fabric. These sensor formulations possess polymethyl methacrylate (PMMA), which enhances the coating stability to only 8% color loss due to wear compared to 18–25% without PMMA, while maintaining the inherent ability of diacetylene-component formulations to detect radiation as well as gas or liquid phase analytes. This represents a significant step toward the use of diacetylene-based sensing formulations for wearable sensing. In the future, the form of spray-on sensor materials demonstrated here may find use in wearable sensing applications for detection of cumulative exposure to UV radiation, hydrogen peroxide vapors, or solvent exposure. We expect trends toward applications toward other wearable sensors for environmental monitoring given the well-known customizability in target response of diacetylene-containing monomers by modifying their headgroup chemistry.

## 1. Introduction

Attention to wearable sensors, including flexible coatings and textile-based wearables, has risen owing to their potential for non-invasive, on-demand detection [1,2,3]. The need for long-term wearability has led to clever designs for self-powered wearable sensors [4,5]. Recently developed spray-on diacetylene-containing sensors might offer an avenue with absolutely no power requirements, having demonstrated capabilities in the colorimetric detection of various stimuli including liquid- or gas-phase analytes, as well as radiation, depending on their formulation [6,7,8]. An attractive feature of color-change-based sensors is their simple readout [9,10]; however, quantitative analysis can be challenging for colorimetric sensing at low target concentrations, given the limitations in human vision [11]. Other colorimetric sensors have been developed for the detection of lactate and glucose via sweat analysis [12,13], volatile organic compounds [14], and for real-time detection and tracking of ions such as Na^+^, Ca^2+^, and K^+^, among others [15].

Nonetheless, sprayable materials have benefits in being able to conform to irregular surface topologies, which enhances their potential for use in wearable sensors and diagnostics [16]. Successful realization of this new class of spray-on materials for wearable sensing will ultimately require the coated sensors to remain on the substrate to which they are deposited. Unwanted removal or color change due to wear as well as any resulting color transfer highlights an existing problem that must be overcome for practical use of these spray-on materials in wearable sensing. Recent studies working on the incorporation of sensing moieties into wearables for the detection of organic targets have often neglected to characterize the impact of mechanical wear, among other human and environmental factors [17,18]. As a surface coating-based sensor, it is equally important to ensure that the sprayed sensing material does not exhibit false-positive signals due to non-target stimuli from regular handling and use. Similarly, avoidance of modifications to coating formulations that could inactivate sensing mechanisms in the coatings is critical to prevent false-negative signals.

While spray-on diacetylene-containing formulations were only recently established [6], implementations of other forms of diacetylene-containing systems, such as thin films and fibres, have previously made use of a variety of supportive polymer additives such as polyvinyl alcohol and polyvinylidene difluoride [19,20,21,22,23,24]. Polydiacetylenes are a class of conjugated polymers consisting of highly ordered backbones and sidechains that are easy to design and alter. Their unique optical properties make them the perfect candidates for biosensors for the detection of physical and chemical stimuli. Polymer-based sensors have a several advantages, such as higher binding efficiency, ease of fabrication, and better stability when compared to other sensor systems such as nanoparticle-based sensors or small-molecule-based sensors [25]. Polymethyl methacrylate (PMMA) has been proven to be a highly flexible polymer with good mechanical stability and high transparency in the visible spectrum [26,27]; thus, we propose that incorporating polymethylmethacrylate (PMMA) as a polymer blend with diacetylene-containing sensor formulations will enable improved wear resistance while retaining the stimuli-responsive behavior of the spray-on sensors. Following from this, the main aim of the present study is to provide an assessment of the wear characteristics for diacetylene-containing spray-on sensor formulations as blends with PMMA. In this work, we focus primarily on the diacetylene-containing amphiphile formulations composed of a constant amount of the stimuli-responsive component, 10,12-pentacosadiynoic acid (herein referred to as PCDA), with distinct concentrations of added PMMA. To investigate the wear properties of our sprayable coatings, we utilized variations of crocking test methods to assess coating loss, transfer of the coating, and retention of the coating’s stimuli response. In addition to changing the PMMA concentration, the extent of UV polymerization time on the diacetylene-containing component of the coating was also varied in order to determine any impact of polymerization on the wear resistance. In this work, we present the extent of color loss from the coating as well as the amount of color fastness to the crock meter squares based on the digitally captured images of the spray coating and the crock meter squares, respectively. RGB image values were converted to CIELAB color space values of luminosity which were measured and compared from the image at the areas of applied crocking vs. uncrocked regions from the same sample serving as reference. We found through our investigation that incorporating the PMMA into spray-on sensing formulations coated onto cotton fabric revealed noticeable improvements in wear resistance, reduction in color transfer, and prevention of false-positive color changes from wetting.

## 2. Materials and Methods

### 2.1. Sample Preparation for Coating the Various Surfaces Used in Wear Testing

In this technique, 20 mg/mL PCDA formulations in methylene chloride carrier solvent were prepared with 0, 8.5, 17, or 34 mg/mL PMMA and sprayed using an airbrush to coat strips of paper, cotton fabric, polyester fabric, and felt fabric substrates (unless otherwise stated all reagents were purchased from Sigma Aldrich Inc., St. Louis, MO, USA). In detail, 10,12-pentacosadiynoic acid (PCDA) amphiphile monomers were prepared by dissolving in methylene chloride. This solution was then passed twice through cotton compressed in a Pasteur pipette that served as a filter, to separate any polymerized particles from the monomer components. After filtering, the purified solution was transferred to a round bottom flask, wherein the methylene chloride was evaporated using a rotary evaporator at 50 °C, 700 mbar (Heidolph Instruments GmbH & Co, Schwabach, Germany). The white-colored powder of the monomeric PCDA was transferred to a dark, amber vial to prevent polymerization and stored at 4 °C. For generation of samples for testing and quantifying the wear caused by dry crocking, a stock solution of the diacetylene amphiphile was prepared by dissolving 20 mg of PCDA in 1 mL of methylene chloride. The stock PCDA solution was sonicated for 1 min using a water bath sonicator (Daihan Scientific Co., Wonju, South Korea) to dissolve any large particles that may have been present in the solution. Samples from different test surfaces were obtained by cutting 14 cm × 3 cm pieces to be tested with a crockmeter. The different test surfaces that were chosen for crocking were paper, cotton fabric, felt fabric, and polyester fabric. This area was airbrushed with the diacetylene-containing solution and left to dry until all the solvent evaporated. Methylene chloride was used as the carrier solvent to dissolve polymethyl methacrylate (PMMA). The PCDA + PMMA formulations were prepared by dissolving 8.5 mg/mL, 17 mg/mL, 34 mg/mL PMMA in methylene chloride by bath sonication and vortexing, and, for each of these concentrations, incorporated 20 mg/mL PCDA by addition of the dry, purified PCDA monomer into the dissolved PMMA, followed by bath sonication and vortexing unless specified otherwise.

### 2.2. Processing of the Spray-On Formulations

**Figure 1:**
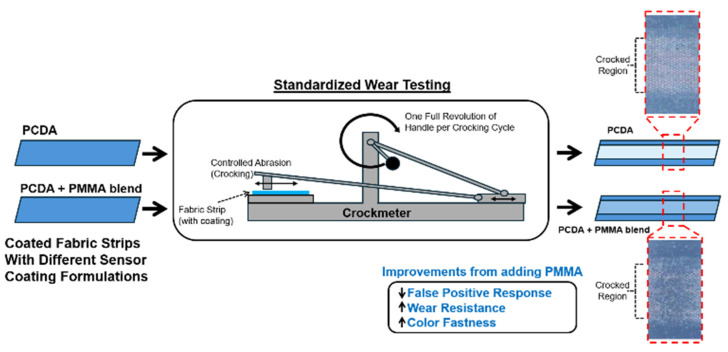
Overview of our study in utilizing PMMA to enhance the wear resistance of PCDA-based, spray-on sensor coatings with assessment after implementing a crockmeter for providing controlled abrasion of coated fabrics.

The freshly prepared PCDA solutions, as well as PCDA- and PMMA-containing solutions, were prepared in methylene chloride with proportions described in detail in the previous section. For spray processing of the formulations, a NEO BCN siphon-feed dual-action airbrush was used. Specifically, the freshly prepared samples were loaded via the reservoir attachment and sprayed by delivery of air with an Iwata Ninja Jet Airbrush Compressor (ANEST Iwata-Medea Inc., Portland, OR, USA). This uniform processing allowed the formulations to be evenly spray-coated onto the surfaces. The substrates used for testing included a grade 1 Whatman filter paper, a 100% cotton cloth, felt fabric, and cloth made from polyester. The substrate was cut into 14 cm × 3 cm strips, and a 3 cm area in the center was airbrushed with exactly 1 mL of our given formulation to provide a homogeneous coating on the surface of the substrate. The formulations were sprayed by holding the airbrush nozzle at a 90° angle and at an approximate distance of 12 cm from the surface of the substrate. The solution was applied by spraying with a side-to-side motion and air-dried for several minutes to ensure complete evaporation of the solvent. As described in the following section and shown schematically in Figure 1, a crockmeter was used to provide controlled cycles of rubbing of the spray-coated surfaces with a crockmeter square to standardize the applied forces, with the resistance to wear assessed by the amount of color loss (by assessing the coating color) and color fastness (by assessing the color transfer to the crockmeter square used for rubbing).

### 2.3. Testing and Quantifying Wear Caused by Dry Crocking of Diacetylene Coating on Various Surfaces

For testing the wear allowance of the polymer formulation on a given substrate, the polymer coating was rubbed using 10 cycles of dry crocking with a crockmeter (AATCC Crockmeter TND006, Hanchen Instruments, Zhengzhou, China). The test substrate was fastened on the specimen holder of the crockmeter, and a 100% cotton AATCC Crockmeter Square (Testfabrics Inc., West Pittston, Pennsylvania, PA, USA) was fastened on the finger of the crockmeter. Each cycle of rubbing with a crockmeter consists of one back-and-forth motion of rubbing the surface. This test was carried out on coating sprayed onto four different substrates: filter paper, cotton cloth, felt fabric, and polyester cloth. The diacetylene coated surfaces were exposed to UV irradiation at 254 nm for 5 s, 10 s, 30 s, and 1 min. These different UV irradiation times were examined in this experiment to test the effects of longer UV irradiation time points on the extent of polymerization of the diacetylene amphiphiles. Initial colors of these spray-coated surfaces were recorded by taking a digital photograph before dry crocking. After crocking was carried out, another digital photograph was taken to record the loss of polymer coverage and to quantify the wear caused by the crockmeter.

Image analysis was carried out using ImageJ software (version ImageJ 1.54j). Red, green, and blue (RGB) color values for the polymer coatings were extracted using the Analyze plugin provided in the ImageJ 1.54j software. The extracted RGB values were then converted to CIELab color space values of L*, a*, and b*. CIELab color space is a close representation of the visual perception of color. Blue-channel image data alone is not highly representative of the complete data for color changes that are observed with PCDA-based sensors, and CIELab color space provides a higher degree of distinction between two shades of blue that may be visually indistinguishable. For example, in some cases, the coating also completely comes off, leaving the surface with the original color of the substrate. Thus, the color change was in some cases quantified by the relative change in the Euclidian distance of the CIELab color space for the a*, b*, and 100-L* values. These a*, b*, and 100-L* values were used for the determination of the extent of white-to-blue color change and polymer loss. In contrast, to calculate the color changes from blue-to-red, such as after exposure to solvent, the CIEDE2000 formula was used to determine the ∆E color space distance. This was performed in Microsoft Excel with the ColorTools add-on and is based on the L*, a*, and b* values.

### 2.4. Modifying Wear Resistance of Diacetylene Coating by Inclusion of PMMA

To increase wear resistance of the polydiacetylene-based coating, PMMA was included in the polymer coating formulation. A quantity of 17 mg PMMA was dissolved in 1 mL methylene chloride. A quantity of 20 mg PCDA was weighed and dissolved in the PMMA/methylene chloride solution. This test was carried out on four different surfaces: 100% cotton fabric, paper, polyester fabric, and felt fabric. Three different UV irradiation time points were examined in this experiment to test the efficacy of longer UV irradiation times on the polymer coating. Digital photographs were recorded before and after crocking the surfaces for 10 cycles. The images were analyzed in ImageJ as described previously.

### 2.5. Testing the Effectiveness of Varying Concentrations of PMMA with Diacetylene Coating on Cotton

Various PMMA concentrations were prepared by dissolving 8.5 mg, 17 mg, and 34 mg PMMA in methylene chloride. For each of the concentrations, 20 mg PCDA was dissolved in the PMMA-methylene chloride solutions. We used 100% cotton as a substrate for this experiment. Three different time points of UV irradiation were tested for this experiment: 10 s, 30 s, and 1 min. The cotton substrate was spray-coated with PCDA+PMMA solution with the help of an airbrush. The substrate was then subjected to 10 rounds of dry crocking with a crockmeter. Digital photographs of the experimental setup and the results were recorded before and after crocking the substrates. The images were then analyzed in ImageJ as described previously.

To test the effectiveness of the polymer coating, the 100% cotton substrate was subjected to 10 cycles of dry crocking. PCDA solution was made by dissolving 20 mg of the diacetylene amphiphile in 1 mL of methylene chloride. Similarly, PCDA + PMMA solution was prepared by dissolving 17 mg PMMA in methylene chloride and then dissolving 20 mg PCDA in the same solution. PCDA and PCDA + PMMA polymer solutions were spray-coated on the 100% cotton substrate by airbrushing. The substrate was allowed to air dry. Digital photographs were recorded before crocking. The substrates were subjected to 10 cycles of dry crocking and exposed to UV irradiation at 254 nm for 10 s. Digital photographs were recorded after crocking and UV irradiation to evaluate the relative change in the blue color of the coating.

For testing the retention of solvatochromic properties of the polydiacetylene coating, the PCDA solution was first prepared by dissolving 20 mg in 1 mL of methylene chloride. PMMA-PCDA solution was prepared by dissolving 17 mg PMMA and 20 mg PCDA in 1 mL methylene chloride. We used 100% cotton as a substrate for this experiment. The polymer solution was spray-coated on the substrate using an airbrush. The substrate was irradiated at 254 nm for 10 s, which was then subjected to 10 cycles of dry crocking. The substrate was cut into smaller pieces (approximately 1 cm × 3 cm) and submerged in 100 % ethanol for 1 min, for the substrate exposed to 10 s of UV radiation. Digital photographs were recorded after each step to observe the change in color after each step.

### 2.6. Wear-Testing PCDA-Coated and PCDA + PMMA-Coated 100% Cotton Strips Through Wet Crocking

We cut 100% cotton fabric into 18 cm × 3.2 cm rectangular strips manually with a Fiskars rotary fabric cutter. These sample strips were airbrushed, as described previously, with varying concentrations of PCDA and PCDA formulations containing PMMA. PCDA-containing solution was formulated by dissolving 20 mg PCDA in 1 mL methylene chloride, and PMMA-containing PCDA formulations were prepared by dissolving 8.5 mg/mL, 17 mg/mL, and 34 mg/mL PMMA to form solutions with final concentrations of 25 nM, 50 nM, and 100 nM, with a constant PCDA concentration of 20 mg/mL. After airbrushing the cotton strips, the solvent was allowed to evaporate completely. After drying, the cotton strips were exposed to UV radiation at 254 nm for 5 s, 10 s, and 30 s. Initial colors of these spray-coated surfaces were recorded by taking a digital photograph before wet crocking the samples.

For the wetting step of this process, reverse-osmosis (RO) water is used. One 14 cm × 14 cm plastic weigh boat, procured through Fisher Scientific, is used to weigh the dried, UV-irradiated samples. A baseline mass of the samples is obtained, as the samples must be wetted to 200% of their dry mass for the wet crocking process, according to ASTM D1776. The dry sample is carefully dipped into RO water on the inverse of the coated side to prevent the test coating from undergoing mechanical wear from rubbing. The freshly wetted sample is re-weighed to confirm it has attained a maximum of 200% of its initial mass through wetting. The wetted sample is then subjected to 10 rounds of crocking and left to dry overnight in a cool and dark place, along with its corresponding crockmeter squares. Once dried, digital photographs are recorded once again for analysis of the color changes and removal of the coating from the wear-tested samples.

### 2.7. Investigating the Pattern of Polydiacetylene Coating Removal by Crockmeter Squares During Crockmeter Wear Testing

Two 100% cotton strips of dimensions 18 cm × 3.2 cm were prepared by airbrushing 20 mg purified PCDA in 1 mL methylene chloride solution. Each strip was polymerized by exposure to 254 nm UV radiation for 10 s. Digital images of these irradiated sample strips were recorded before wear testing. One strip was dry crocked for 10 cycles with one crockmeter square. This displays the cumulative behavior of coating removal from the coated strip, as well as cumulative coating transfer to the crockmeter square, and any color changes in the polydiacetylene coating caused from wear. The second strip was also dry crocked for 10 cycles, but with a fresh crockmeter square for each cycle of crocking. This sample was for investigation of coating removal, transfer, and color changes from the cotton strip to show how such changes induced through wear were concentrated or distributed among each of the 10 cycles of dry crocking. After wear testing, digital images of the samples and their respective crockmeter squares were recorded to observe the changes in the coating incurred through wear.

## 3. Results and Discussion

### 3.1. Effect of UV Polymeriztion and Use of PMMA Blends on Wear Resistance of Sensor Coatings

**Figure 2:**
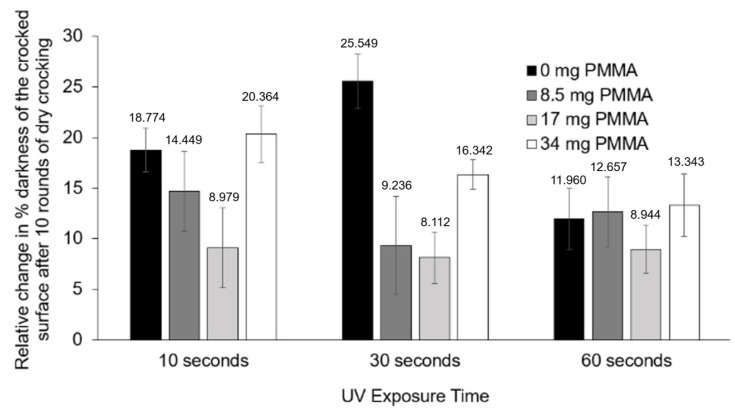
The effect of PMMA concentration and UV polymerization on the amount of coating lost due to crocking was examined. PCDA solutions of 20 mg/mL containing different concentrations of PMMA, and different extents of UV irradiation, were subjected to 10 rounds of dry crocking. Relative change in the % darkness of the coating was calculated by the difference between the (100-L*) values before and after 10 cycles of dry crocking. This difference was then divided by the (100-L*) value before crocking, and the percentage value was obtained.

Initially, we sought to examine a preferred concentration of PMMA within our PCDA-based spray-on sensor formulation that could improve the resistance to removal by wearing/rubbing when coated onto cotton fabric. The results of the experiments seen in Figure 2 revealed that increasing PMMA concentration provided increased wear resistance (to dry crocking) of the coating on cotton for up to 17 mg/mL of PMMA. Testing double that amount of PMMA resulted in poor resistance to wear. This result was consistent across various UV polymerization times for the stimuli-responsive coating. Here, it is important to note that UV polymerization allowed for visualization of the quality of the coating (including any coating loss after crocking) as well as for visualization of any impact of varying UV exposure times on the wear resistance of the coating. We noticed no consistent relationships between UV exposure times and wear resistance; however, a significant improvement using 17 mg/mL PMMA was evident.

### 3.2. Effect of Incorporating PMMA Blends on Color-Fastness of Spray-On Sensor Formulations

**Figure 3:**
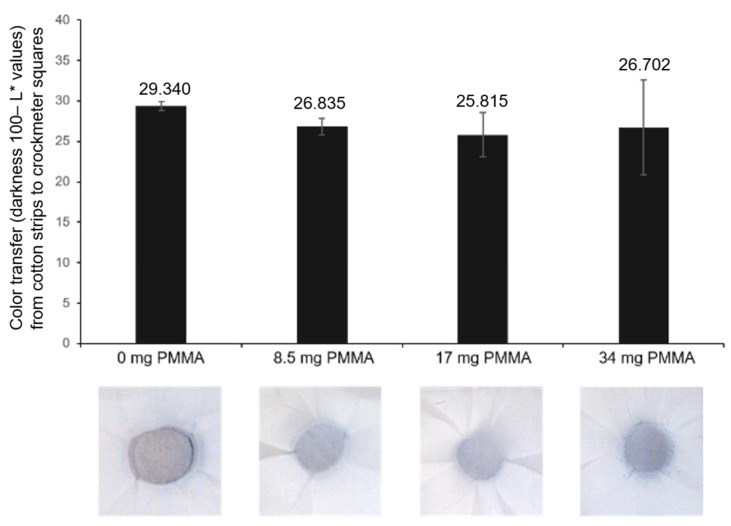
Color fastness (transfer of PCDA from the coatings to the crock meter squares) was examined as a function of PMMA concentration within the spray formulations. The darkness of the transferred color (100−L*) values for each crock meter square are reported, which represent the amount of PCDA coating transferred onto the crock meter square used for rubbing the substrate in each case.

Selecting 17 mg/mL PMMA as the preferred amount of supporting polymer to mitigate coating loss to rubbing, we next examined its application on other substrates. Figure S1 provides the images and color loss data for dry crocking of the PCDA formulations with and without 17 mg/mL PMMA as applied to substrates of cotton fabric, paper, polyester fabric, and felt fabric. The coatings when applied to substrates composed of natural cellulose-based fibers, specifically the paper and cotton fabric, exhibited better resistance to wear from dry crocking. This is expected to be a result of the formulations utilizing methylene chloride carrier solvent, to which synthetically derived substrates may be unstable. As shown in Figure 3, the color-fastness, or resistance to color transfer from the coated substrate to the crock meter squares during dry crocking, was also found to improve for PCDA formulations containing 17 mg/mL PMMA. A supplementary crocking test, Figure S2, in which 10 new crocking squares were sequentially used to assess the color-fastness, similarly revealed that the PMMA component helped to provide enhanced color-fastness of the spray-on PCDA coating.

### 3.3. Examining if PMMA Blend Component Affects the Sensing Capabilities of the Formulations

In addition to wear resistance, it is necessary to also assess if the PMMA component would negatively impact the sensing capabilities of the PCDA coating. Two modes of detection are possible with spray-on diacetylene-containing sensors; thus, we examined both to determine if the stimuli-responsive nature of the formulations remained when adding PMMA (that is to say, avoiding false-negative signals due to adding this component).

**Figure 4:**
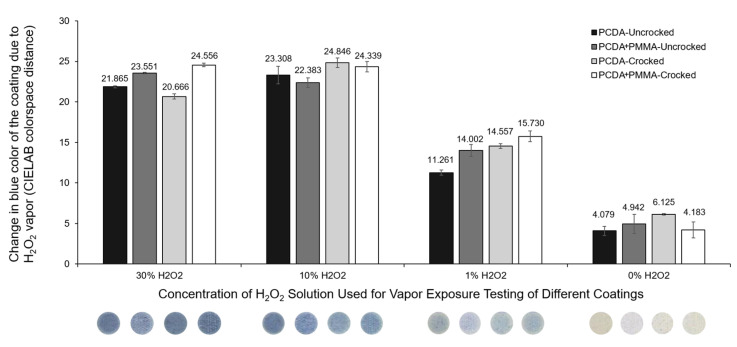
Retention of function of the diacetylene coating to generate a polymerization response upon exposure to H_2_O_2_ vapor was examined. Diacetylene solutions containing different concentrations of PMMA were coated on separate cotton substrates and subjected to 10 rounds of crocking. The crocked and uncrocked samples were exposed to H_2_O_2_ vapor at different concentrations. The color change was measured by calculating the CIELAB color space distance using the a* and b* values of the substrate before and after exposure to H_2_O_2_ vapor.

The first mode of detection we examined was the ability of the coating color to change from white to blue upon polymerization of the PCDA monomer components, wherein the extent of polymerization provides a mechanism for detecting the amount of stimulus to which the sensing material is subjected. The detection of UV radiation was demonstrated above in the prior figures and provided initial evidence that the PMMA does not impact the polymerization-based response mechanism, as the resulting color change was clearly visible. By incorporating a catalytic component (specifically iron cations) in the formulation, we have previously shown it is also possible to detect target gases that can be transformed by the catalyst to generate local radical species that initiate polymerization of the PCDA component to similarly give a white-to-blue color change [7]. As shown in Figure 4, the PCDA coating formulations containing iron catalyst were able to retain their ability to detect the presence of hydrogen peroxide gas when combined with PMMA. In this experiment, we also found that the white-to-blue color response for the coating was still possible even if the coating had previously been subjected to 10 cycles of rubbing with the crockmeter, thereby demonstrating that when adding PMMA, the coating retained function as an effective sensor based on the polymerization mechanism even when subjected to abrasive wear.

**Figure 5:**
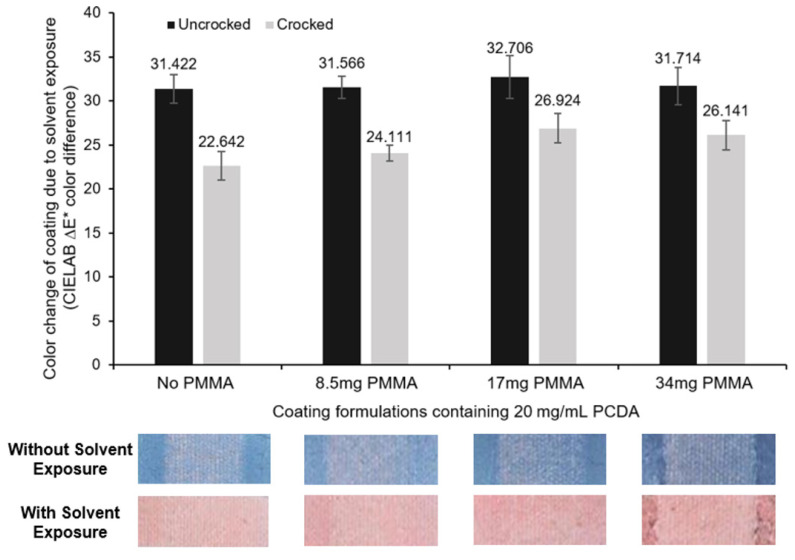
Retention of function of the diacetylene coating to undergo a color change upon exposure to solvent was examined, specifically using 100% ethanol. PCDA formulations with different concentrations of PMMA were coated onto cotton fabric, exposed to UV for polymerization, and subjected to 10 rounds of dry crocking. The samples were then exposed to ethanol, revealing an observable color change. The color change was calculated by applying the CIEDE2000 color-difference formula for ΔE* which uses the L*, a*, and b* values measured for the substrate before and after exposure to 100% ethanol to determine the solvent responsiveness (solvatochromism).

The second mode of detection can occur once the coating composed of adjacent diacetylene-containing amphiphiles is already polymerized to the blue phase. This second mode is an observed blue-to-red color change in response to stimuli which is due to the effective shortening of the conjugation length of the polydiacetylene (PDA) backbone [28,29]. This results from molecular-level rotation of the PDA backbone caused by stimuli-induced rearrangement of the PDA side-chains [30,31]. We demonstrate in Figure 5 that addition of PMMA to the spray-on PCDA formulations did not disturb this stimuli-responsive behavior [6], as we can clearly see the blue-to-red color transition in response to liquid target exposure; specifically, the known solvatochromism behavior to ethanol was retained [31]. Importantly, we also see that the solvent response in the form of a blue-to-red color transition was also maintained in the areas that underwent rubbing with the crockmeter.

### 3.4. Effects of Incorporating PMMA Blend on Wash-Induced Shrinkage Response of Sensor Coating

**Figure 6:**
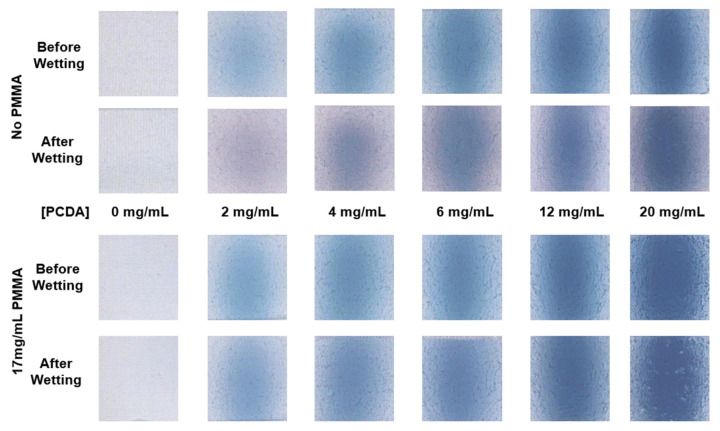
Upon wetting and drying of the polydiacetylene coated cotton strips, a color change was observed to have taken place from blue to purple/pink, depending on the concentration of the polydiacetylene solution in the specific region in the gradient. To investigate this further, decreasing concentration of PCDA were spray-coated onto the cotton fabric strips, and it was observed that at lower concentrations, the color change was more apparent. Upon addition of 17 mg/mL PMMA to this formulation, no change in color was observed, thus retaining its use as a colorimetric sensor for detection of stimuli.

We also examined wet crocking of the cotton fabrics sprayed with PCDA coating formulations with and without PMMA, and once again compared the UV polymerization times of the coatings. The addition of PMMA to the coating formulation did not appear to provide a consistent difference in wear resistance in terms of wet crocking when comparing the different UV polymerization times, as seen in Figure S3. We did, however, find that the wetting of the PCDA spray-coated cotton in water induced a noticeable blue-to-red color change in the PCDA coating alone, particularly when using low concentrations of PCDA in the spray formulation, which can be seen in Figure 6. This false-positive signal is undesirable, and we believe this may have been due to the reported mechanoresponsive capability of diacetylene-containing sensors previously reported [32]. When conducting wetting–drying cycles of the cotton strip, we saw a noteworthy shrinkage in the fabric up to four wash–dry cycles (Figure S4). In contrast, the use of 17 mg/mL PMMA in the PCDA spray formulation resulted in the elimination of this false-positive color change upon wetting, as this response to water would be unwanted for a wearable sensor. We believe that adding the PMMA component provided resistance to this undesired response to water based on a combination of the PMMA hydrophobicity as well as the PMMA mechanical stability, of which both may have contributed to resisting local structural changes during the wetting–drying of the cotton fibers. While we did not quantify it, we did observe a very small but noticeable difference in the stiffness of the coatings incorporating PMMA, which was to be expected.

### 3.5. Examining the Porosity and Uniformity of Coatings on Cotton Fabric

**Figure 7:**
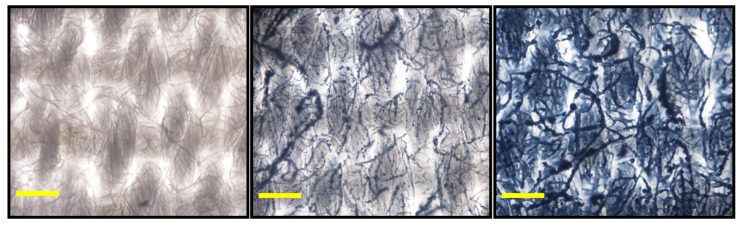
Optical microscopy images of cotton fabric without spray-on sensor coating (**left**), with spray coating formulation of 20 mg/mL PCDA (**middle**), or with spray coating formulation of 20 mg/mL PCDA containing 17 mg/mL PMMA (**right**). Coatings were UV polymerized for 10 s. (Scale bars indicate 0.2 mm.)

Finally, we examined how spray-coating the sensing material onto cotton fabric affected the porosity of the material, and we also examined how uniformly the spray-on sensor coating was applied to the fabric. The porosity was determined by measuring the weight of the dry fabric samples (either uncoated, PCDA coated, or PCDA-PMMA coated) followed by fully saturating the fabric sample with water and weighing the saturated fabric to determine the amount of absorbed water. On average, the porosity was found to be 65.2% for the uncoated cotton fabric, 61.3% for the 20 mg/mL PCDA-coated cotton fabric, and 54.8% for the cotton fabric coated with the 20 mg/mL PCDA formulation containing 17 mg/mL PMMA. These results indicated a small decrease in the porosity when using the coating formulation. An optical microscopy image of the uncoated, PCDA coated, and PCDA/PMMA blend coated cotton fabric is shown in Figure 7. Using the natural fluorescence of the polydiacetylene coatings, samples of coated fabrics were assessed for coating uniformity, as determined by examining the coefficient of variation in the polydiacetylene fluorescence intensity across 16 regions of each sample (with each 2.4 mm-by-1.6 mm region corresponding to the field of view under 4× magnification). This measurement confirmed that the PCDA spray coating exhibited 95% uniformity, while the PCDA/PMMA blend spray coating was similar, exhibiting 96% uniformity. Comparing the images in Figure 7, it is clear that there is an even distribution of material coating across the cotton fabric, and perhaps most interesting is the degree to which the addition of PMMA to the formulations appeared to allow better integration with the cotton fibers. This enhanced integration with the fibers of the cotton fabric may be a contributing factor to the improved resistance to wear for the PCDA formulations containing PMMA.

## 4. Conclusions

In summary, we demonstrate here the incorporation of PMMA into formulations of PCDA spray-on sensors, wherein a key outcome we found is that addition of PMMA could provide significant enhancement in the wear resistance properties of the spray-on sensing materials to dry crocking and similarly improved color fastness. In addition, adding the PMMA component to the PCDA formulations did not weaken the stimuli-responsive behavior of the coating compared to PCDA alone, suggesting that the PMMA added to the formulation does not result in false-negative signals. That is to say, the liquid- and gas-phase response to target exposure previously identified for the PCDA formulations was retained even when adding the PMMA blend to enhance wear resistance. An interesting revelation through the study, however, was that the formulation with PCDA alone sprayed onto cotton fabric exhibited a false-positive color change when submerged in water; however, formulations with the addition of the PMMA component were able to eliminate the unwanted false-positive color change due to submersion in water. Furthermore, in this study, we found that all formulations spray-coated onto cellulose-based substrates (cotton or paper) would outperform coatings onto synthetic substrates in terms of wear resistance. In looking closely at the wear resistance, we believe an optimal PMMA content exists near 17 mg/mL, since wear resistance greatly diminished at the higher PMMA concentration of 34 mg/mL. With these improvements in the enhanced wear resistance, retention of the color-change responsiveness to UV radiation, as well as liquid and gas target stimuli (ethanol and hydrogen peroxide vapor respectively), and the elimination of false-positive response to wetting–drying-induced fabric shrinkage, it is clear that the PMMA component imparted significant benefits to the spray-on sensor formulations. Areas primed for future efforts in the use of spray-on wearable sensors are expected to be for environmental monitoring, given that the visual color change intensifies with cumulative exposure of target compounds or UV radiation. We hope this demonstration of the feasibility for wear resistance in spray-on sensor coatings inspires future work in exploring polymer blends with diacetylene-based sensing components for even further improvements in this new application area of sprayable sensors.

## Data Availability

Data supporting this article have been included as part of the Supplementary Information.

## Supplementary Materials

The following supporting information can be downloaded at: https://www.mdpi.com/article/10.3390/s24216925/s1, Figure S1: Dry crocking of PCDA or PCDA+PMMA spray formulations coated onto different substrates; Figure S2: Wear resistance of polydiacetylene coated cotton strips via the use of one crockmeter square for 10 cycles of crocking vs. using a new crockmeter square after each round of crocking; Figure S3: Effect of different UV times and presence of PMMA on coating loss due to wet crocking; Figure S4: Shrinkage of cotton strips as a function of wetting-drying cycles.
