# Supplementary material for: Assessing Wear Characteristics of Sprayable, Diacetylene-Containing Sensor Formulations
Source: Sensors (Basel). 2024 Oct 29;24(21):6925. doi: 10.3390/s24216925 (PMC11548148; doi:10.3390/s24216925)
Supplement: Supplementary file 1 [file sensors-24-06925-s001.zip › sensors-3198208-supplementary.pdf]

Supplementary Materials

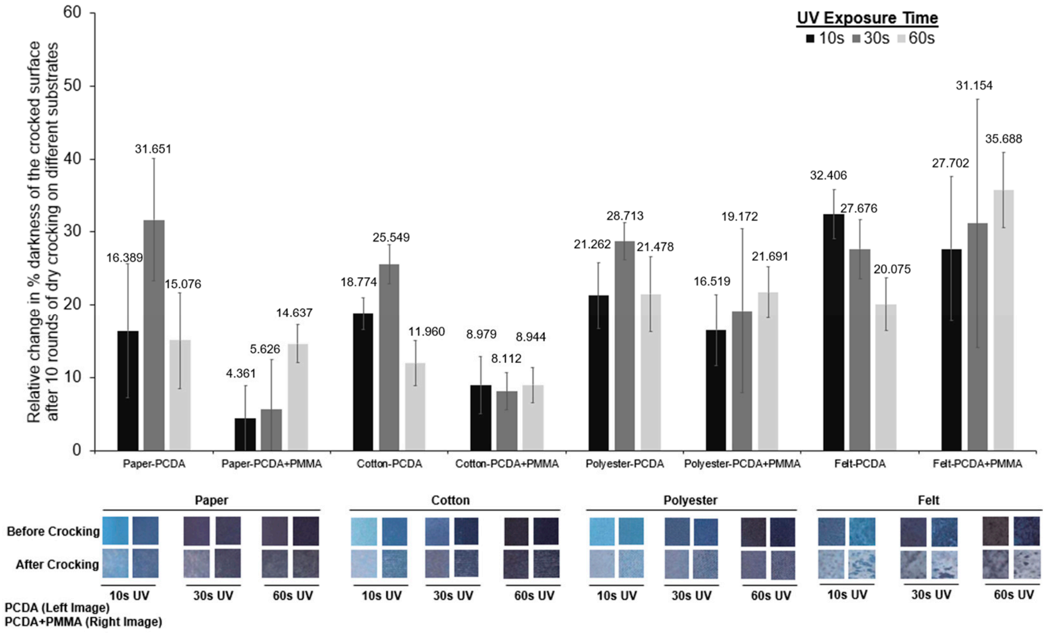

Figure S1. Dry crocking of PCDA or PCDA+PMMA spray formulations coated onto different substrates (Paper, Cotton, Polyester, and Felt) provided examination of the colour response of the spray-on polydiacetylene formulation used in wear testing for the different substrates. Relative change in the % darkness of the coating was calculated by the difference between the (100-L*) values before and after 10 cycles of dry crocking. This difference was then divided by the (100-L*) value before crocking and the percentage value was obtained. The formulation the PCDA spray coating was 20mg/mL PCDA in methylene chloride carrier solvent, while the PCDA+PMMA spray coating was 17mg/mL PMMA and 20mg/mL PCDA in methylene chloride carrier solvent.

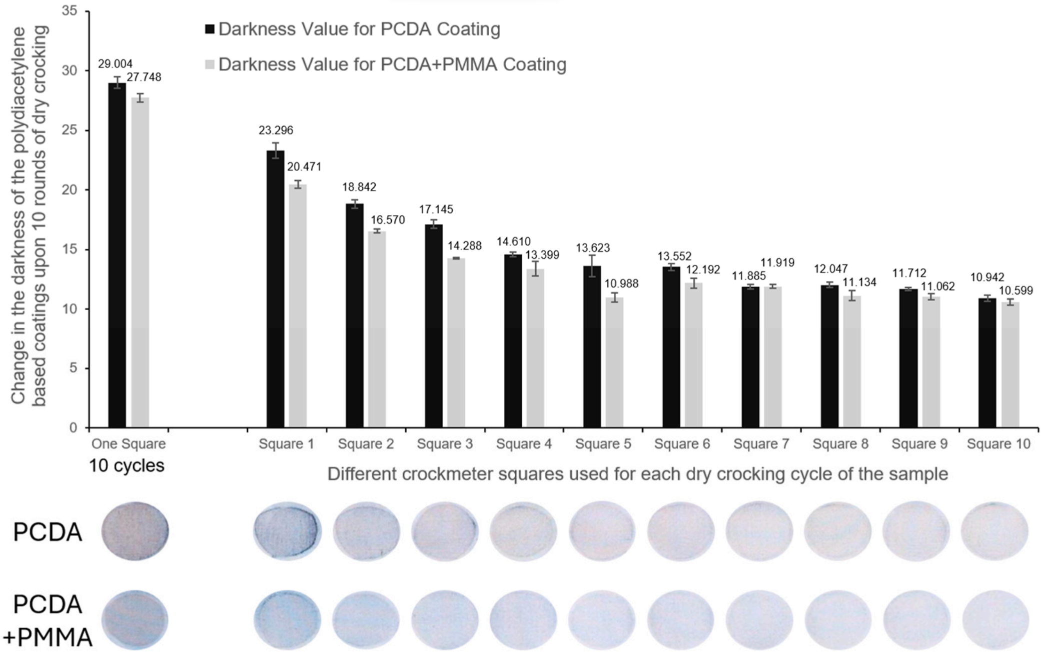

Figure S2. Demonstration of the distribution of wear on polydiacetylene coated cotton strips via the use of one crockmeter square for 10 cycles of crocking vs using one crockmeter square per round of crocking. (100-L*) values for each crockmeter square are reported here to visualize the amount of polydiacetylene coating transferred to the crockmeter (colour fastness) in each case. The formulation the PCDA spray coating was 20mg/mL PCDA in methylene chloride carrier solvent, while the PCDA+PMMA spray coating was 20mg/mL PCDA and 17mg/mL PMMA in methylene chloride carrier solvent.

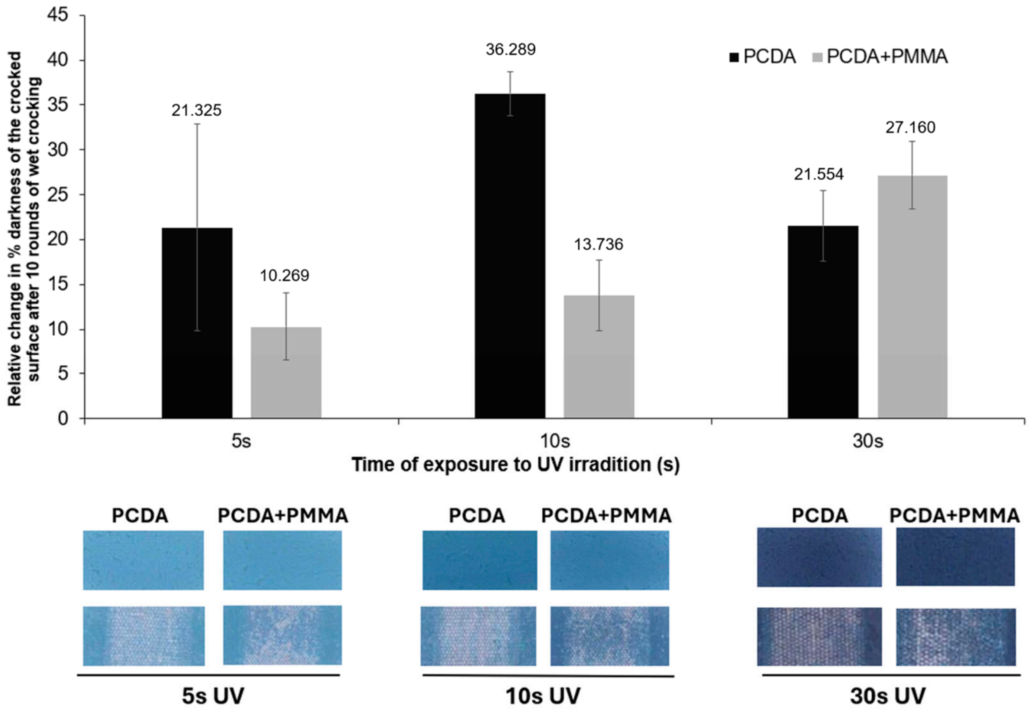

Figure S3. Effect of different UV times and presence of PMMA on coating loss due to wet crocking. Cotton strips coated with polydiacetylene solutions were subjected to crocking to quantify the wear caused to the coating while it is still wet. Addition of PMMA did not enhance the wear resistance as much as it did in the case of samples that were dry crocked. The relative change in the % darkness of the coating was calculated by the difference between the (100-L*) values before and after crocking. This difference was then divided by the (100-L*) value before crocking and the percentage value was obtained. The formulation the PCDA spray coating was 20mg/mL PCDA in methylene chloride carrier solvent, while the PCDA+PMMA spray coating was 20mg/mL PCDA and 17mg/mL PMMA in methylene chloride carrier solvent.

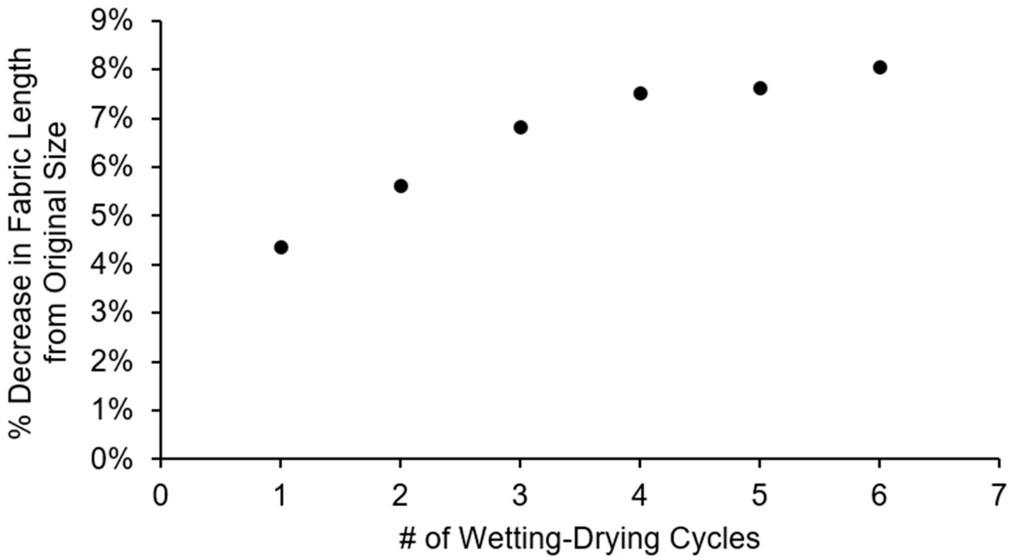

Figure S4. Shrinkage of cotton strips as a function of wetting-drying cycles. Cotton strips were subjected to multiple cycles of wetting and drying, using an oven at 60 °C to calculate the % shrinkage of the strip from its original length.
